# A novel role for the SUMO E3 ligase PIAS1 in cancer metastasis

**DOI:** 10.18632/oncoscience.27

**Published:** 2014-03-31

**Authors:** Shorafidinkhuja Dadakhujaev, Carolina Salazar-Arcila, Stuart J. Netherton, Amrita Singh Chandhoke, Arvind Kumar Singla, Frank R. Jirik, Shirin Bonni

**Affiliations:** ^1^ Department of Biochemistry and Molecular Biology, University of Calgary, Calgary, Alberta, Canada; ^2^ Southern Alberta Cancer Research Institute, University of Calgary, Calgary, Alberta, Canada; ^3^ McCaig Institute for Bone and Joint Health, University of Calgary, Calgary, Alberta, Canada

**Keywords:** Cancer metastasis, SUMO E3 ligase, PIAS1, three-dimensional cultures

## Abstract

Tumor metastasis contributes to the grave morbidity and mortality of cancer, but the mechanisms underlying tumor cell invasiveness and metastasis remain incompletely understood. Here, we report that expression of the SUMO E3 ligase PIAS1 suppresses TGFβ-induced activation of the matrix metalloproteinase MMP2 in human breast cancer cells. We also find that knockdown of endogenous PIAS1 or inhibition of its SUMO E3 ligase activity stimulates the ability of TGFβ to induce an aggressive phenotype in three-dimensional breast cancer cell organoids. Importantly, inhibition of the SUMO E3-ligase activity of PIAS1 in breast cancer cells promotes metastases in mice *in vivo*. Collectively, our findings define a novel and critical role for the SUMO E3 ligase PIAS1 in the regulation of the invasive and metastatic potential of malignant breast cancer cells. These findings advance our understanding of cancer invasiveness and metastasis with potential implications for the development of biomarkers and therapies in breast cancer.

## INTRODUCTION

Tumor cell invasiveness and metastasis pose a major obstacle in the treatment of cancer [1-3]. Although mechanisms that transform cells into tumor cells have been characterized [4], the regulation of tumor cell invasiveness and metastasis remains poorly understood. Epithelial mesenchymal transition (EMT) represents a fundamental cellular process that is thought to contribute to epithelial tumor cell invasiveness and metastasis [5-8]. Therefore, determining the role of regulators of EMT in the control of the malignant features of epithelial tumors including their invasiveness and metastasis represents a worthwhile strategy in gaining novel insights in cancer biology.

EMT comprises the loss of apical-basal polarity and of cell-cell adhesions resulting from the downregulation or mislocalization of epithelial markers such as E-cadherin and the expression of mesenchymal proteins such as N-cadherin [6, 7]. In addition, the actin cytoskeleton is reorganized during EMT from cortical to stress fiber-like morphology. A critical functional outcome of EMT is the increased motility of cells, which may provide a basis for the significance of EMT in cancer cell behavior [9, 10].

The cytokine transforming growth factor β (TGFβ) plays a central role in the control of EMT [11-13]. TGFβ potently induces EMT in distinct cell types including mammary gland epithelial cells [12, 13]. TGFβ-induced EMT may play a critical role in normal tissue morphogenesis during development [10, 14, 15]. In addition, TGFβ-induced EMT may contribute to the ability of TGFβ to promote tumor progression and metastasis in later stages of neoplastic disease in epithelial tissues [5, 8, 16, 17].

Among cell-autonomous mechanisms, the SUMO E3 ligase PIAS1 has emerged as a critical regulator of EMT [18]. PIAS1 contains a RING domain, which mediates the interaction of PIAS1 with the SUMO E2 conjugating enzyme Ubc9 [19-21]. PIAS1 acts as a SUMO E3 ligase by specifying target proteins for SUMO conjugation by Ubc9. The sumoylation activity of PIAS1 was recently found to suppress TGFβ-induced EMT in non-transformed epithelial cells [18]. Accordingly, TGFβ induces EMT in non-transformed epithelial cells via the degradation of PIAS1, suggesting that PIAS1 represents a critical regulator of TGFβ-induced EMT in normal epithelial cells [18]. These findings have raised the fundamental question of the role of the SUMO E3 ligase PIAS1 in the control of cancer cell invasiveness and metastasis.

In this study, we identify a novel function for PIAS1 in cancer metastasis. In gain of function studies, we find that PIAS1 acts in a SUMO E3 ligase-dependent manner to suppress the ability of TGFβ to promote activation of the matrix metalloproteinase 2 (MMP2) and associated invasive behavior of breast cancer cells. In a three-dimensional Matrigel culture model system, we find that TGFβ promotes the disorganization and invasive behavior of breast cancer cell derived-organoids. TGFβ reduces the abundance of E-cadherin and PIAS1 in breast cancer cell-derived organoids. Consistently, loss and gain of function studies demonstrate that PIAS1 acts in a SUMO E3 ligase-dependent manner to counteract the ability of TGFβ to induce the invasive and aggressive phenotypes of breast cancer cell-derived organoids. Remarkably, PIAS1 also acts in a SUMO E3 ligase-dependent manner to suppress the ability of breast cancer cells to metastasize to bone in a murine xenograft model. Collectively, our findings reveal that the SUMO E3 ligase PIAS1 regulates TGFβ-induced cancer cell invasion and metastasis.

## RESULTS

### The SUMO E3 ligase PIAS1 suppresses TGFβ-induced MMP2 activity and cell invasiveness in mammary carcinoma cells

**Figure 1:**
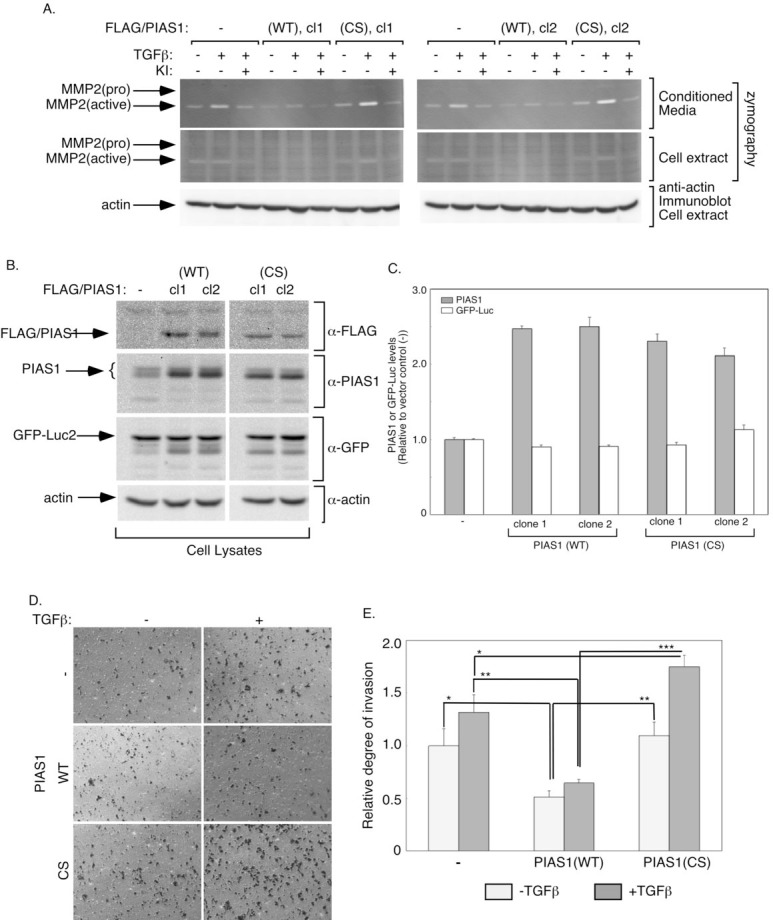
PIAS1 acts in a SUMO E3 ligase-dependent manner to suppress TGFβ-induced MMP2 activity and invasiveness of MDA-MB-231 breast cancer cells A. Conditioned medium (upper panels) or cellular extract (middle panels) of stable MDA-MB-231 cells expressing GFP-luciferase fusion protein alone (-), or together with wild type (WT) (clones 1 and 2), or SUMO E3 ligase mutant (CS) (clones 1 and 2) PIAS1, incubated in low serum alone or together with 100 pM TGFβ alone or together with SB4321542 (KI) for 48 h, were subjected to gelatin zymography followed by Coomassie staining. Cellular extracts were also immunoblotted using an actin antibody, serving as loading control (lower panels). Approximate MW of pro-MMP2 (72 kDa) and active MMP2 (62 kDa) were determined by the relative mobility of protein markers. B. Lysates of stable MDA-MB-231 cells expressing control, PIAS1 (WT), or PIAS (CS) as described in A were subjected to FLAG, PIAS1, GFP, and actin immunoblotting, with the latter serving as a loading control. C. Each column in the bar graph represents the mean -/+ SEM of actin-normalized PIAS1 and GFP-luciferase for each stable cell line expressed relative to the respective parameter of the vector control values from three independent experiments. D and E. Untreated (light grey) or 48 h-TGFβ treated (dark grey) of stable MDA-MB-231 cells expressing control, PIAS1 (WT), or PIAS1 (CS) as described in A, were subjected to a transwell invasion assay using Matrigel-coated inserts. After 12 h incubation at 37°C, cells in the upper chamber were removed and cells invading into the underside of the inserts were fixed, stained with crystal-violet, and eight randomly chosen fields were visualized at 2X (Figure S1) and 10X (Figure 1D) objectives, and counted. The experiments were performed in duplicates and repeated three-independent times. The data in the graph (E) represent the mean +/-SEM of cells appearing on the underside of Matrigel-coated inset of the transwell from three independent experiments. Data from clones 1 and 2 of PIAS1 (WT) or PIAS1 (CS) clones were combined. *, **, or ***, refers to P < 0.05, P< 0.01, or P< 0.001, respectively (ANOVA). TGFβ potently promoted the invasion of each of the control (-) and PIAS1 (CS)-expressing cells (p=0.009, t-test), and modestly increased the invasion of PIAS1 (WT) expressing cells (p=0.034, t-test).

To investigate the role of the SUMO E3 ligase PIAS1 in the invasive and metastatic behavior of epithelial tumor cells, we used human MDA-MB-231 breast cancer cells. These breast cancer cells display evidence of epithelial mesenchymal transition (EMT) in standard two-dimensional cultures [22, 23]. The activation of matrix metalloproteinases (MMP) represents a key feature of EMT that may also play a critical role in tumor invasiveness and metastasis [24, 25]. In gelatin zymography analyses, we detected the activity of the enzyme MMP2 in lysates and conditioned medium of MDA-MB-231 breast cancer cells (Figure 1A). Exposure of MDA-MB-231 cells to TGFβ further induced MMP2 activation in these cells, an effect that was blocked by the TGFβ type I receptor kinase inhibitor (Figure 1A), indicating that activation of TGFβ signaling stimulates the activation of MMP2 in these cells.

We assessed the effect of expression of PIAS1 on the ability of TGFβ to augment MMP activation in MDA-MB-231 breast cancer cells (Figure 1A-1C). We found that expression of PIAS1 suppressed the ability of TGFβ to induce MMP2 activation in MDA-MB-231 cells (Figure 1A). In contrast, expression of a SUMO E3 ligase inactive mutant of PIAS1, owing to replacement of Cysteine 350 with serine (PIAS1 (CS)), enhanced TGFβ-induced activation of MMP2 in MDA-MB-231 breast cancer cells (Figure 1A) [18, 26]. These results suggest that the SUMO E3 ligase PIAS1 regulates TGFβ-induced MMP2 activation in breast cancer cells.

We next determined the role of PIAS1 in the invasive behavior of breast cancer cells. Consistent with the function of PIAS1 in regulating TGFβ-induced MMP2 activation, we found that expression of wild type PIAS1 suppressed the ability of TGFβ to confer an invasive behavior in MDA-MB-231 breast cancer cells (Figures 1D, 1E, S1). By contrast, expression of the SUMO E3 ligase deficient PIAS1 (CS) mutant augmented TGFβ-induced invasiveness in MDA-MB-231 cells (Figures 1D, 1E, S1). These findings suggest that PIAS1 regulates the invasive property of MDA-MB-231 breast cancer cells.

### PIAS1 acts in a SUMO E3 ligase-dependent manner to suppress TGFβ-induced disruption of breast cancer cell-derived organoids

**Figure 2:**
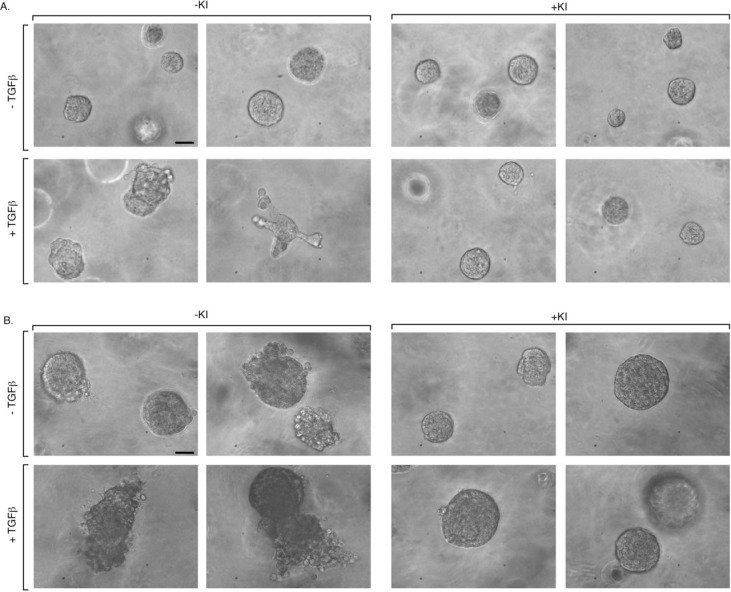
TGFβ induces disorganization and budding of MDA-MB-231 breast cancer cell-derived organoids Human MDA-MB-231 breast cancer cells were grown in Matrigel in three-dimensional cultures in ultralow attachment 96-well plates (Materials and Methods). Cultures were either left untreated or incubated with the TGFβ type I receptor kinase inhibitor SB431542 (KI), TGFβ alone or together with the inhibitor. Three-dimensional multicellular structures were monitored at regular intervals by light microscopy. Representative images of 5-day (A) and 10-day (B) three dimensional-derived organoids captured by digital camera phase contrast microscopy are shown from an experiment that was repeated two independent times. Scale bar indicates 50 μm..

The finding that PIAS1 regulates TGFβ-induced MMP2 activation and invasiveness in breast cancer cells led us next to determine the role of PIAS1 in the invasive and metastatic behavior of breast cancer. We first approached this question using a model of breast cancer cell organoids. To establish breast cancer cell-derived organoids, we employed a three-dimensional culture system in which MDA-MB-231 breast cancer cells were grown in the context of matrix support to provide an environment that more closely reflects the *in vivo* setting. The MDA-MB-231 cell-derived organoids were monitored at distinct time intervals by phase microscopy. We found that MDA-MB-231 cells formed filled spheres with different degrees of outward protrusions upon longer periods in culture (Figure 2A, 2B). Because non-transformed mammary epithelial cells form spheres with lumens in three-dimensional cultures that mimic mammary gland tissue [27-29], these results suggest that MDA-MB-231 cell-derived organoids reflect distortion of the normal structure of mammary epithelial cell-derived tissue. This interpretation is consistent with EMT-like behavior of MDA-MB-231 cells in standard two-dimensional cultures.

We asked whether TGFβ alters the morphology of the MDA-MB-231 cell organoids. We found that TGFβ induced further deformation of MDA-MB-231 cell-derived structures in three-dimensional cultures. TGFβ induced the appearance of substantial protrusions and budding of the MDA-MB-231 cell-derived structures (Figure 2A, 2B). The TGFβ-induced effect was blocked upon incubation of the three dimensional cultures with the TGFβ receptor inhibitor SB432154 (Figure 2A, 2B), indicating that TGFβ-induced effects in the three dimensional cultures are specific and occur through activation of the TGFβ receptor. Consistent with these results, TGFβ triggered the downregulation of E-cadherin in three-dimensional cultures of MDA-MB-231 cells (Figure S2). Taken together, these data suggest that the three dimensional cultures of MDA-MB-231 cells represent a suitable model system for characterization of the mechanisms that underlie the malignant behavior of breast cancer cells.

**Figure 3:**
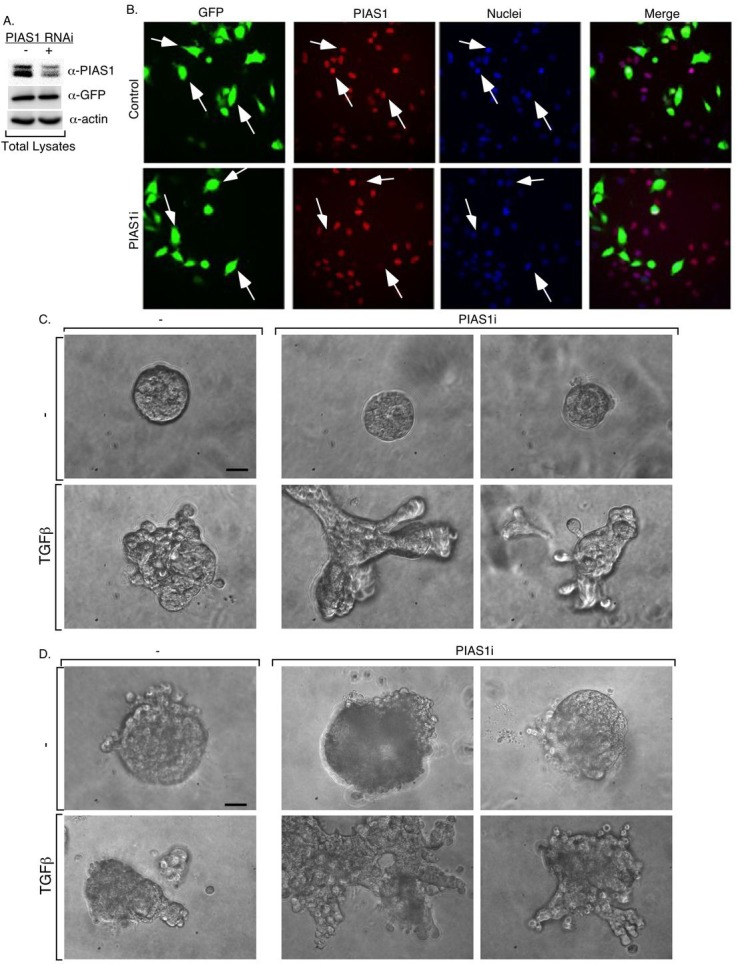
Knockdown of endogenous PIAS1 enhances TGFβ-induced disorganization of MDA-MB-231 breast cancer cell-derived organoids MDA-MB-231 cells transiently transfected with a control RNAi vector or with a combination of two vectors each expressing short hairpin (sh) RNA targeting a unique sequence in PIAS1 for knockdown, were subcultured into 60 mm dishes (A) or in multiple wells of a 96-well plate (B). The RNAi vectors also coexpressed GFP to visualize transfected cells. Transfected cells were subjected to PIAS1, GFP and actin immunoblotting and quantitative analyses for PIAS1 (A) or to PIAS1 indirect immunofluorescence and nuclear Hoechst staining and visualization of PIAS1 (red), GFP (green) and nuclei (blue) by fluorescence microscopy at 20X magnification (B). Arrows indicate examples of cells transfected with the control (-) or PIAS1 (PIAS1i) RNAi vectors. PIAS1 RNAi triggers efficient and specific knockdown of PIAS1. Representative micrographs of 5-day (C) and 10-day (D) live three-dimensional cultures of MDA-MB-231 cells transfected with control (-) and PIAS1 RNAi (PIAS1i) vectors as described in A and B that were left untreated or incubated with TGFβ as described in Figure 2. Experiments were repeated 3 independent times. Scale bar indicates 50 μm.

We next determined the function of PIAS1 in TGFβ-regulation of MDA-MB-231 breast cancer cell-derived organoids. We induced the acute knockdown of PIAS1 in MDA-MB-231 cells using RNAi. We used two short hairpin RNAs (shRNAs) targeting distinct sequences within PIAS1, which individually or in combination led to efficient knockdown of exogenous PIAS1 in 293T cells (Figure S3A). In immunoblotting or immunocytochemical analyses, the two PIAS1 shRNAs triggered efficient knockdown of endogenous PIAS1 in MDA-MB-231 cells (Figures 3A, and S3B). Importantly, in analyses of morphology of MDA-MB-231 cell-derived structures, we found that knockdown of PIAS1 substantially enhanced the ability of TGFβ to induce outward growth, budding, and branching of MDA-MB-231 cell-derived organoid structures (Figure 3C, 3D). These data suggest that endogenous PIAS1 suppresses the ability of TGFβ to induce the aggressive behavior of breast cancer cell-derived organoids.

**Figure 4:**
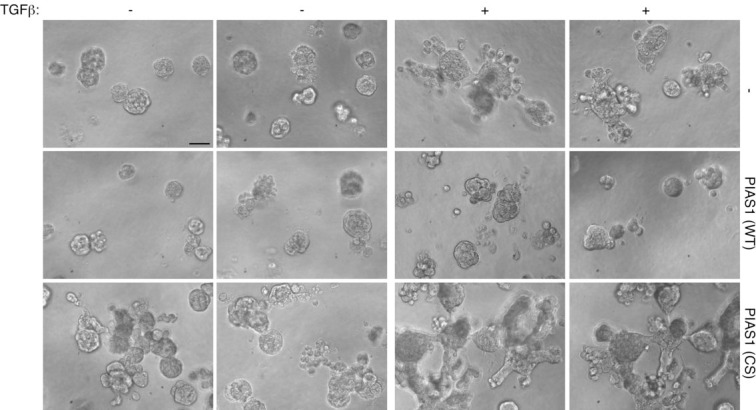
The SUMO E3 ligase PIAS1 inhibits TGFβ-induced disorganization of MDA-MB-231 breast cancer cell-derived organoids Matrigel-embedded three-dimensional cultures of MDA-MB-231 cells stably expressing PIAS1 (WT) or SUMO E3 ligase mutant PIAS1 (CS), or vector were prepared in ultralow attachment 96-well plate and left untreated or incubated with TGFβ for 2 weeks. 10-day live three-dimensional multicellular structures were visualized and imaged as described in Figure 2. Scans of two representative fields are shown taken from each of MDA-MB-231 stable cells in the absence and presence of TGFβ from an experiment that was repeated five independent times. Scale bar indicates 50 μm.

In a complementary line of experiments, we characterized the effect of stable expression of PIAS1 in MDA-MB-231 cells on the morphology of the organoids in three-dimensional cultures. Expression of wild type PIAS1 maintained an organized MDA-MB-231 multicellular spherical structure and reduced the proportion of organoids with protrusions (Figure 4). Importantly, the expression of wild type PIAS1 suppressed the ability of TGFβ to induce deformation of MDA-MB-231 cell-derived organoids including the formation of protrusions (Figures 4 and S4A-S4C). By contrast, we found that expression of the SUMO E3 ligase PIAS1 (CS) mutant increased the proportion of organoids harboring protrusions and triggered the growth and branching of large protrusions in the organoids (Figures 4 and S4-S4C). In addition, the expression of PIAS1 (CS) augmented the ability of TGFβ to induce an aggressive phenotype in the MDA-MB-231 cell-derived organoids (Figure 4). Notably, the expression of wild type or CS mutant of PIAS1 had little or no effect on the population growth rate of MDA-MB-231 cells in the three-dimensional cultures (Figure S4D). In other experiments, incubation of MDA-MB-231 cells in three-dimensional cultures with the TGFβ receptor antagonist suppressed the ability of PIAS1 (CS) to disrupt the MDA-MB 231 organoids and promote their invasiveness (Figure S5A). Consistently, TGFβ induced the downregulation of endogenous PIAS1 in MDA-MB-231 cells, an effect that was reversed by co-incubation with the TGFβ receptor kinase inhibitor (Figure S5B). Collectively, our data suggest that PIAS1 acts in a SUMO E3 ligase-dependent manner to suppress the ability of TGFβ to promote an aggressive invasive behavior in MDA-MB-231 cancer cell-derived organoids.

### PIAS1 suppresses breast cancer metastasis in vivo

**Figure 5:**
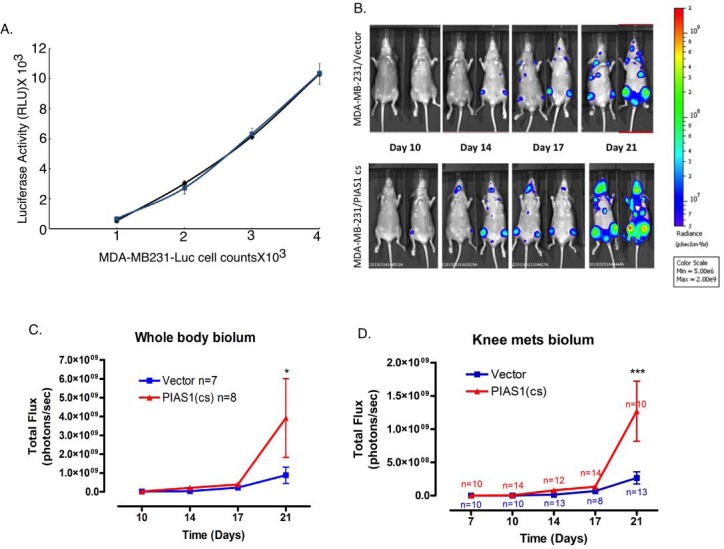
PIAS1 suppresses MDA-MB-231 breast cancer cell-derived metastasis in mice *in vivo* A. Stable clones of the MDA-MB-231-Luc expressing the SUMO E3 ligase mutant PIAS1 (CS) (blue symbols and line) or transfected with the vector control (black symbols and line) were seeded in a 96-well plate at increasing concentrations and subjected to luciferase assay one day later. Linear regression indicated an R2 of 0.98 for the relationship between cell number and luciferase activity for both control and PIAS1 (CS) clones. B. Representative ventral bioluminescence images of mice taken at 10, 14, 17 and 21 days post intracardiac injection of vector control or PIAS1 (CS) stably expressing MDA-MB-231-Luc breast cancer cells. C. Whole body bioluminescence for the control and PIAS1 (CS) expressing breast cancer cells. D. Knee bone metastasis analysis. *, or ***, refers to P < 0.05, or P< 0.001, respectively (ANOVA).

The novel finding that PIAS1 acts in a SUMO E3 ligase-dependent manner to suppress TGFβ-induced breast cancer cell invasiveness using cellular, molecular, and organoid readouts raised the fundamental question of whether PIAS1 might regulate breast cancer metastasis *in vivo*. To address this question, we employed a mouse model in which tumor metastasis is monitored by bioluminescence imaging. We used MDA-MB-231 breast cancer cells (MDA-MB-231-Luc) stably expressing GFP-Luciferase [30]. To investigate the role of PIAS1 as a SUMO E3 ligase on metastatic tumor and growth potential *in vivo*, we determined the effect of expression of the SUMO E3 ligase PIAS1 (CS) mutant in the MDA-MB-231-Luc cells on their ability to form osseous metastases. The PIAS1 (CS) mutant acts in a dominant negative manner to inhibit endogenous PIAS1-regulation of MDA-MB-231 breast cancer cell invasiveness in standard and three-dimensional cultures (Figures 1, 4, S4, and S5). Notably, the expression of PIAS1 (CS) had little or no effect on the bioluminescence of MDA-MB-231-Luc cells *in vitro* (Figure 5A), suggesting that inhibition of PIAS1-dependent sumoylation activity may not affect the proliferation of these cells. We introduced MDA-MB-231-Luc cells expressing the PIAS1 (CS) mutant or the corresponding vector-control transfected control cells into the arterial circulation of NIH-III mice via the intra-cardiac injection route [30]. The efficiency of injection of cells and time-dependent tumor development and growth were determined using live animal bioluminescence imaging [30]. As expected, vector-control transfected MDA-MB-231 control cells metastasized to bone as reflected by the appearance of tumor-derived bioluminescence in one or both knees in the NIH-III mice (Figure 5B). Remarkably, the expression of PIAS1 (CS) shortened the latency to initial bioluminescence signal detection and also augmented the rate of growth of the MDA-MB-231-derived tumors in mice as determined by measurement of whole body or knee photon emission rates (Figure 5C, D). These data suggest that blockade of the SUMO E3 ligase activity of endogenous PIAS1 promotes the rate of MDA-MB-231 breast cancer cell growth in bone. Collectively, our findings define a novel function for the SUMO E3 ligase PIAS1 in the regulation of breast cancer invasiveness and metastasis.

## DISCUSSION

In this study, we have discovered that the SUMO E3 ligase PIAS1 is a critical regulator of breast cancer invasion and metastasis. PIAS1 suppresses TGFβ-dependent activation of the matrix metalloproteinase MMP2 and the invasiveness of breast MDA-MB-231 cancer cells. We have also found that PIAS1 inhibits in a SUMO E3 ligase-dependent manner the ability of TGFβ to promote the disruption and invasive behavior of MDA-MB-231 breast cancer-derived organoids in three-dimensional cultures. Finally, disruption of the SUMO E3 ligase activity of PIAS1 in MDA-MB-231 breast cancer cells enhances the rate of development of bone metastasis in mice following intracardiac injection of the breast cancer cells. Collectively, our findings define a critical role for the interplay of PIAS1 and TGFβ signaling in the regulation of breast cancer metastasis.

The finding that PIAS1 inhibits breast cancer invasion and metastasis bears significant implications for our understanding of the biology of epithelial tumors. Correlating with the ability of PIAS1 to suppress breast cancer invasiveness and metastasis, PIAS1 inhibits TGFβ-induced activation of the matrix metalloproteinase MMP2 as well as the induction of outgrowths from MDA-MB-231 breast cancer cell-derived organoids. These findings suggest that PIAS1 suppresses breast cancer metastasis through the inhibition of TGFβ signaling. Because TGFβ is thought to promote tumor metastasis via the induction of EMT-like behavior in tumor cells at the primary site of tumor formation, our observations suggest that PIAS1-suppression of tumor metastasis occurs via its ability to inhibit TGFβ-induced EMT. Consistent with this interpretation, PIAS1 inhibits the morphological and molecular features of EMT in epithelial cells [18].

In future studies, it will be important to determine how PIAS1 suppresses the growth of breast cancer cell-derived skeletal metastases, a process that shows some dependency on latent TGFβ released from bone as a result of osteoclastic activity [31, 32]. PIAS1 induces the sumoylation of the transcriptional regulator SnoN and thereby suppresses TGFβ-induced EMT [18, 26]. Therefore, it will be interesting to assess whether the sumoylation of SnoN impacts breast cancer metastasis. In future studies, it will be also important to determine whether PIAS1 function intersects with other regulators of breast cancer metastasis including the transcriptional regulators Zeb1/2 and Twist, which are both induced by TGFβ signaling and promote tumor metastasis [5, 8].

The three-dimensional organoid model system used in this study facilitated the identification of the SUMO E3 ligase PIAS1 as a regulator of breast cancer invasion and metastasis. The organoid model helped illuminate the interplay between PIAS1 and TGFβ signaling in controlling the invasive behavior of the breast MDA-MB-231 cells in the context of an environment that more closely reflects the *in vivo* conditions. Thus, our study highlights the utility of the three-dimensional culture method for the identification of novel genes that regulate tumorigenesis and metastasis and may thus lead to the discovery of novel biomarkers and therapeutic targets.

In conclusion, we have uncovered the SUMO E3 ligase PIAS1 as a novel regulator of breast cancer invasion and metastasis. PIAS1 represents a cell-autonomous mechanism that regulates TGFβ-induced breast cancer progression. Our study advances our understanding of mechanisms that control cancer invasion and metastasis and bear implications for the development of biomarkers and therapies in breast cancer.

## MATERIALS AND METHODS

### Plasmids

The pCAGiP/FLAG/PIAS1 (WT) and (CS) expression vectors, where the gene product of interest and the puromycin resistance marker are coexpressed from an internal ribosomal entry site (IRES) containing-bicistronic transcript, were generated as described elsewhere [18]. The PIAS1 RNA interference vector encoding PIAS1 short hairpin RNAs (shRNAs) and enhanced green fluorescent protein (GFP) under the control of the U6 and CMV promoters, respectively, was described previously [33, 34]. PIAS1 RNA interference (RNAi) plasmids were constructed using the pU6/CMV/ enhanced green fluorescent protein (GFP) expression control vector, with PIAS1 short hairpin RNAs (shRNAs), and GFP under the control of U6 and CMV promoters, respectively. Two shRNAs-expressing constructs were generated to target distinct regions in PIAS1 mRNA as follows: PIAS1i-1, 5′ GGATCATTCTAGAGCTTTAAT 3′, PIAS1i-2, 5′GGGTTTGTCCTGTCTGTG ATAA 3′. The plasmids were confirmed by restriction digests and/ or DNA sequence analyses (University of Calgary Core Sequencing Facility).

### Mice

5-6 week old, female, nude (athymic)-beige (NIH-III) mice were purchased from Charles River Laboratories, maintained at 22°C in a 12 hr light and dark cycle with *ad libitum* access to chow and water. Experiments were conducted in compliance with Canadian Council on Animal Care guidelines, and with ethics approval from the University of Calgary's Animal Care Committee.

### Cells and culturing condition

The human embryonic kidney 293T cells and human breast cancer MDA-MB-231 cells were confirmed to be free of pathogenic *Mycoplasma* strains by a PCR-ELISA kit (Roche Applied Biosciences). MDA-MB-231 cells expressing EGFP-Luciferase 2 (MDA-MB-231-Luc), generated as described elsewhere [30], were grown in selection medium-containing Dulbecco's modified Eagle medium (DMEM; Invitrogen, Grand Island, NY) supplemented with 10% heat-inactivated fetal bovine serum (FBS), 1.2 mg/ml geneticin (Invitrogen) at 37°C in a 5% CO2 humidified atmosphere, and were routinely passaged every 2-3 days. To generate PIAS1-stably expressing MDA-MB-231-Luc cells, the control pCAGiP vector (containing a puromycin resistance gene) alone, or pCAGiP vector containing wild-type PIAS1 (WT) cDNA, or a SUMO E3 ligase inactive PIAS1 (C350S) cDNA, were linearized and transfected into cells using Lipofectamine 2000, as per the manufacturer's instructions [18, 35]. Transfected cells were selected using 1μg/mL puromycin-(Invitrogen) containing-complete medium.

### Cell extract preparation and Immunoblotting

Cell were incubated for 10 minutes at 4°C in TNTE lysis buffer (50 mM Tris, 150 mM NaCl, 1 mM EDTA, 0.5% [v/v] Triton-X-100) containing protease inhibitors (1 mM phenylmethylsulfonyl fluoride (PMSF) and 10 μg/ ml pepstatin A (Sigma), 100 μg/ml benzamidine chloride (Calbiochem), and 1 mg/ml trypsin inhibitor, 10 μg/ ml antipain, 50 μg/ml aprotinin and 10 μg/ml leupeptin (Roche Applied Biosciences)), and phosphatase inhibitors (10 mM sodium pyrophosphate and 25 mM sodium fluoride (EM Sciences), and 1 mM sodium orthovanadate (Alfa Aesar)). Lysates were centrifuged at 15,000X g for 10 minutes at 4°C, and small aliquots were subjected to protein concentration determination using Bradford-based protein assays (Bio-Rad Laboratories). Cell extracts were resolved by sodium dodecyl sulfate-polyacrylamide electrophoresis (SDS-PAGE) and were transferred onto nitrocellulose membrane (Bio-Rad Laboratory). The blots were incubated with mouse anti-FLAG (Sigma), rabbit anti-PIAS1 (Epitomics) or rabbit anti-actin (Sigma), as the primary antibody and HRP-conjugated donkey anti-mouse or anti-rabbit IgG (Jackson Laboratories) as secondary antibodies, followed by ECL and signal detection using a VersaDoc 5000 Imager (Bio-Rad Laboratories). Densitometry was performed using Quantity One software (Bio-Rad Laboratories).

### Luciferase determination

Luciferase activity in MDA-MB-231-Luc cells stably expressing PIAS1 or transfected with control vector was assessed using a commercially available luciferase kit (Promega) and a luminometer (Orion Microplate Luminometer, Berthold Detection Systems) as a measure of protein abundance of this reporter. Briefly, serial dilutions of 1000 to 4000 cells per well were plated in 96-well plates in triplicates and subjected to a commercial firefly luciferase assay kit as described previously [36]. Each experimental condition was carried out in triplicate, and independent experiments were conducted at least 4 times.

### Gelatin Zymography

MDA-MB-231-Luc cells stably expressing PIAS1 (WT) or PIAS1 (CS), or transfected with the control vector, were seeded at 5×10^4^ cells/ml in 10cm dishes in 10% FBS-containing DMEM, and switched the next day to serum-free DMEM in the absence or presence of 100 pM TGFβ (R&D), alone or together with 10 μM of the TGFβ type I receptor inhibitor SB431542 (Sigma). Two days post-treatment, conditioned media (CM) and cells were collected separately, centrifuged at 5000 rpm for 5 min, lysed in 2x Tris-Glycine SDS sample buffer with incubation at room temperature for 10 minutes. 20 to 30 μg of each of CM and cell lysates were subjected to gelatin zymography where protein mixtures were resolved using 7.5% SDS-polyacrylamide gel-containing 0.1% porcine skin-derived gelatin (Sigma) [37, 38]. Following electrophoresis, gels were washed 3 × 20 minutes at room temperature in Zymogram Renaturing Buffer (2.5% Triton X-100) to remove SDS, and incubated in Zymogram Developing Buffer (ZDB) (50 mM Tris-HCl pH 7.6 buffer-containing 0.15 M NaCl, 5 mM CaCl2 and 0.05% NaN3) for 30 min at room temperature. Gels were incubated in fresh ZDB at 37°C for at least 24 hours, stained with 0.25% Coomassie Brilliant Blue R-250 (Bio-Rad) in a methanol: acetic acid: water (2.5: 1: 6.5) solution, and de-stained with a 4% ethanol + 8% acetic acid solution. Stained gels were scanned using a VersaDoc 5000 Imager (Bio-Rad Laboratories).

### *In vitro* transwell invasion assays

Invasion of MDA-MB-231-Luc cells was assayed using Matrigel-coated transwell polycarbonate filters (24-well insert, pore size 8 μm; BD Biosciences). Inserts were equilibrated by adding 0.5 ml serum-free DMEM to the upper and lower chambers and incubating at 37°C for 2hr. After gentle aspiration of the equilibration medium, the upper chambers were coated with 40 μl of Matrigel (300 μg/μl) followed by incubation at 37°C for 1hr. Cells harvested after overnight serum deprivation were seeded at 105 cells in 0.5 ml of serum-free DMEM on the upper Matrigel-coated chamber. 10% FBS-containing DMEM, alone or together with 100 pM TGFβ was added to the lower chamber. Following a 12 h incubation at 37°C, non-adherent cells were removed by washing cell layers twice with phosphate buffered saline (PBS), followed by removal of adherent cells on the upper layer of the insert by gentle scraping with a cotton tip applicator. Cells that had invaded the underside of the inserts were fixed with 100% methanol for 10 minutes at −20°C and stained with 0.5% crystal violet dye (EMD Millipore) for 1hr at room temperature. Images of crystal-violet-stained invading cells in eight randomly chosen fields of each lower chamber were captured by light microscopy at 10X objective (Olympus IX70) coupled to a digital camera. Images were also captured at 2X objective (Figure S1). Total cell counts in the 8 fields was obtained using Image J program (National Institutes of Health Image). Each experiment was done in triplicates with several independent repeats.

### Immunocytochemistry and Cellomics-based Analyses

MDA-MB-231 cells transfected with green fluorescence protein (GFP)-expressing control or PIAS1 RNAi plasmids were seeded in 96-well plate at 105 cell/ ml. Two days later, cells were 4% paraformaldehyde-fixed (Fisher), permeabilized with 0.2% Triton-X (Sigma), blocked using 5% bovine serum albumin (Roche) and 5% calf serum (VWR) containing PBS. Cells were then subjected to indirect immunocytochemistry for PIAS1 using rabbit-anti PIAS1 (Epitomics), as primary antibody, and a Cy3-conjugated anti-rabbit IgG (Jackson Laboratories), as secondary antibody, and Hoechst-DNA-staining using bisBenzimide H33342 trihydrochloride (Invitrogen to visualize their nuclei. Fluorescence images were acquired using the Cellomics Kinetic Scan Reader that is equipped with Carl Zeiss Axiom x microscope and a charge coupled device (CCD) digital camera [18, 35]. The spot detector bioapplication was used to quantify PIAS1 fluorescence intensity where each cell was identified by nuclear stain and GFP signal (transfected cells). Each condition was carried out in triplicates, and the immunofluorescence data were averaged from a minimum of 2000 cells per well. The intensity of PIAS1 immunofluorescence in transfected MDA-MB-231 cells was expressed as a percent of the PIAS1 immunofluorescence in the control vector-transfected cells (Figure S3B).

### Three-dimensional cultures

Three-dimensional culture of MDA-MB-231 cells were prepared by first coating 96-well flat-bottom, ultra low attachment plates (BD Biosciences) or 8-well glass chambers (Millipore) with 50 μl or 75 μl, respectively, of 30% diluted growth-factor-reduced Matrigel (3 mg/ ml) (BD Bioscience) in complete medium (DMEM-containing 10% FEBS, penicillin, streptomycin and amphotericin B (Invitrogen) and incubating for 1 h in a 5% CO2 humidified incubator at 37°C to form a 1 mm thick 30% Matrigel bed. The 30% diluted Matrigel-containing medium was kept on ice prior to transfer to the appropriate tissue culture vessel. 50 μl or 75 μl of MDA-MB-231 cell suspensions in 30% Matrigel final concentration was carefully layered on top of the Matrigel bed in the 96-well or 8-chamber slide, respectively. The following day and at every third day, three-dimensional cultures received fresh complete medium alone, or with 100 pM TGF-β, 10 μM TGFβ type kinase inhibitor SB431542, alone or together. Differential interference contrast (DIC) images of the 5-day and 10-day live three-dimensional multicelluar structures were captured using light microscopy at 20 X objective (Olympus IX70). Three-dimensional cell cultures were then fixed subjected to indirect immunocytochemistry to visualize E-Cadherin and PIAS1 using rabbit anti-E-Cadherin antibody (Cell Signaling), or rabbit anti-PIAS1 antibody, respectively, as a primary antibody, followed by cy2-conjugated or cy3-conjugated anti-rabbit antibody as the secondary. Three-dimensional cultures were also incubated with the nucleic acid-fluorescent dye Hoechst to visualize nuclei. Fluorescence microscopy was used to capture images of multicellular colonies at 40 X objective (Zeiss Axiovert 200M microscope).

### Breast Cancer Cell Metastasis Model

To generate metastases, 5–6 week old female NIH-III mice were anesthetized by intraperitoneal (i.p.) injection of ketamine (100 mg/kg) and xylazine (6 mg/ kg), and then 2×10^5^ MDA-MB-231-Luc cells stably expressing resistant marker alone or together with the SUMO E3 ligase inactive PIAS1 (CS) suspended in 100 μl of PBS were injected into the left ventricle of each mouse as previously described (30). Successful intra-cardiac injections were confirmed by immediate whole body bioluminescence imaging (showing systemic distribution of bioluminescence signal), and development of metastases was monitored at different days by bioluminescence imaging.

### Bioluminescence imaging

Bioluminescence imaging was performed as described previously [30]. Mice were administered *D*-luciferin (Gold Bio Technology) at a dose of 150 mg/ kg in PBS by i.p. injection, and anesthetized with 1.5–2% isofluorane for 10–12 min prior to imaging. Animals were then placed onto the warmed stage inside of the IVIS light-tight chamber and anesthesia was maintained with 1.5–2% isofluorane. For the image acquisition, the cooled charge coupled device (CCD) camera of Xenogen IVIS Lumina system (Caliper Life Sciences) was used; mice were imaged in both the dorsal and ventral positions. Imaging parameters were f/stop 1, bin 4, field of view 12.5 cm, and exposure times ranged from 20 s to 2 min, depending on the strength of the tumor-derived photon emission rates. Results were analyzed using Living Image 3.0 software (Caliper Life Sciences). Signal intensity was quantified as the total photons/s within a uniform region of interest positioned over specific tumor sites (as well as over the entire body) during the data post-processing, with any background bioluminescence subtracted out.

### Statistical analyses

All data were plotted as mean-/+SEM. Statistical analysis was performed using t-test or ANOVA followed by Student Newman Keuls test (InStat) or Mann-Whitney *t*-test (GraphPad Prizm 4.0 Software) as post hoc tests. Values of *P*<0.05 were considered statistically significant.

## SUPPLEMENTARY FIGURES


