# Supplementary material for: A novel role for the SUMO E3 ligase PIAS1 in cancer metastasis
Source: Oncoscience. 2014 Mar 31;1(3):229–40. doi: 10.18632/oncoscience.27 (PMC4278292; doi:10.18632/oncoscience.27)
Supplement: Supplementary file 1 [file oncoscience-01-0229-s001.pdf]

A novel role for the SUMO E3 ligase PIAS1 in cancer metastasis - Dadakhujaev et al

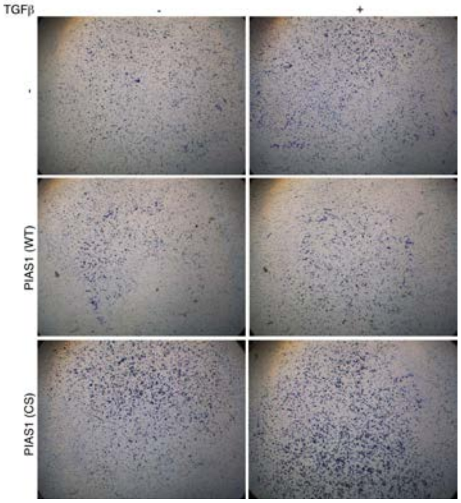

Figure S1. Related to Figure 1.
Light microscopy images of crystal violet stained untreated or TGFβ-incubated MDA-MB-231 breast cancer cells stably expressing wild type (WT) PIAS1 or a SUMO E3 ligase inactive PIAS1 (CS) in which Cysteine 350 was mutated into serine, or transfected with the empty vector (-) appearing on the underside of the Matrigel-coated insert of the transwell taken at 2Xobjective. Refer to Figure 2 E and F and methods for details.

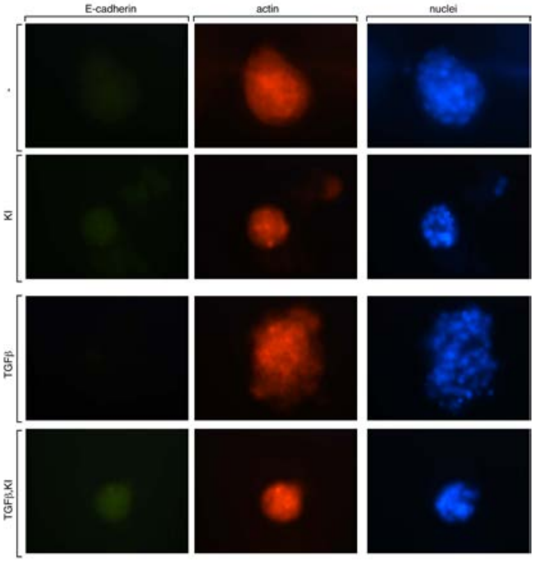

Figure S2. Related to Figure 2.
Fluorescence images of 10 day in gel-paraformaldehyde-fixed three-dimensional cultures of MDA-MB-231 breast cancer cells left untreated or incubated with the TGFβ type I receptor kinase inhibitor SB431542 (KI) or TGFβ as described in Figure 2, and subjected to indirect immunofluorescence to

visualize E-cadherin (green), and incubation with the fluorescently labeled phalloidin to detect actin (red) and the nucleic acid-fluorescent dye Hoechst 33342 to identify nuclei (blue). Images were taken at 40Xobjective. Experiment was repeated 2 independent times. TGFβ signaling reduces the abundance of E-cadherin in MDA-MB-231 cell-derived organoids.

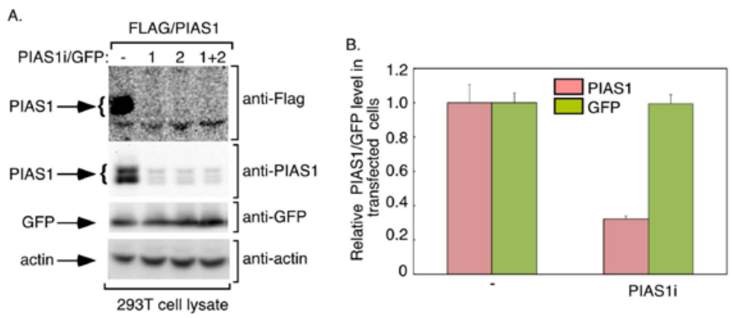

Figure S3. Related to Figure 3

A. Lysates of 293T cells transfected with a FLAG/PIAS1 expression vector together with a control RNAi vector (-), or PIAS1 RNAi-1 (PIAS1i1) plasmid, PIAS1 RNAi-2 (PIAS1i2) plasmid, individually or in combination, were subjected to immunoblotting using anti-FLAG, anti-PIAS1, anti-GFP, and anti-actin antibodies. GFP (green fluorescence protein) is expressed as part of the control and PIAS1 RNAi plasmids (Materials and Methods). PIAS1 RNAi plasmids produced efficient knockdown of exogenous PIAS1. B. MDA-MB-231 cells transiently transfected with a control RNAi vector or with the combination of PIAS1 RNAi-1 and RNAi-2 plasmids (PIAS1i), were subcultured in multiple wells of a 96-well plate, and subjected to PIAS1 indirect immunofluorescence and nuclear Hoechst staining and visualization for PIAS1, GFP and nuclei by fluorescence microscopy using the Cellomics Kinetic Scan Reader (Figure 2B and Materials and Methods). Bar graph represents the mean-/+ SEM of PIAS1 (red) or GFP (green) fluorescence intensity in transfected cells expressed relative to the control RNAi vector transfected cells. PIAS1 RNAi induced significant knockdown of endogenous PIAS1 in MDA-MB-231 cells (P<0.001, t-test).

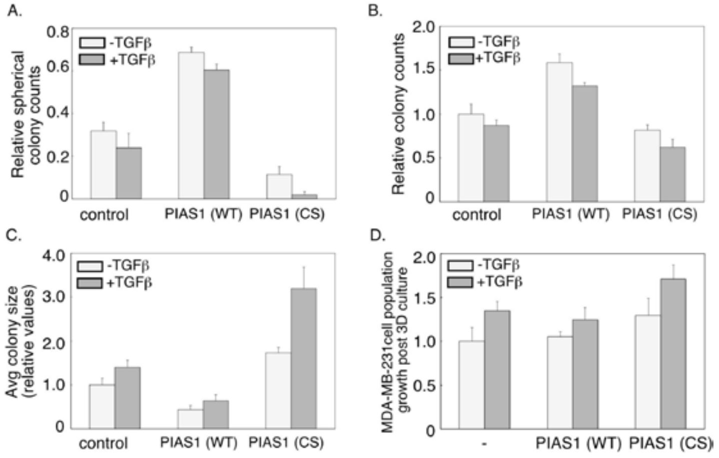

Figure S4. Related to Figure 4.

Quantitative analyses of relative (A), total counts (B), and average size (C) of three dimensional spherical organoids derived from MDA-MB-231 breast cancer cells stably expressing vector control, PIAS1 (WT) or PIAS1 (CS) as determined by Motic 2.0 software. Data shown are the mean (-/+SEM) from 5 independent experiments including the one shown in Figure 4. In A, PIAS1 (WT) increased, while PIAS1 (CS)

decreased the ratio of spherical organoids as compared to the control (ANOVA, $p<0.001$). In B, PIAS1 (WT) decreased, while PIAS1 (CS) increased average colony size as compared to the control (ANOVA, $P <0.01$). For C, PIAS1 (WT) expressing cells showed more colonies as compared to control and PIAS1 (CS) (ANOVA, $p<0.01$). D. Cell counts of untreated or TGFβ-treated three-dimensional cultures of MDA-MB- 231 cells. Cells were extracted from three-dimensional cultures and counted by light microscopy. Data represent the mean (-/+SEM) of cell counts from three independent experiments.

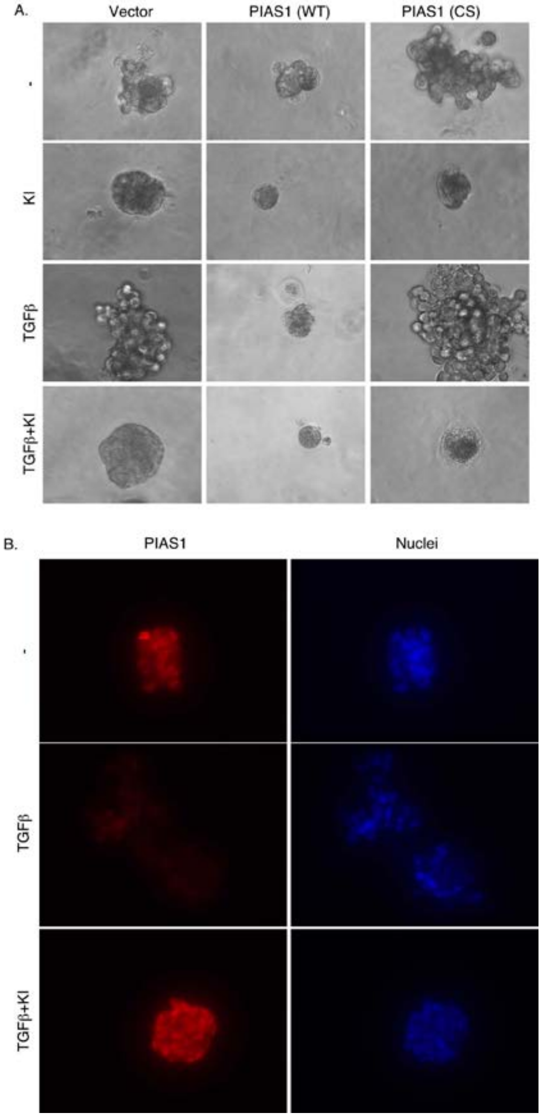

Figure S5. Related to figure 4.
A. DIC scans of 10 day-three-dimensional organoids derived from MDA-MB-231 breast cancer cells expressing PIAS1 (WT) or PIAS1 (CS), or transfected with the vector control were left untreated or incubated with the TGFβ type I receptor kinase inhibitor SB431542 (KI), TGFβ, alone or together and then subjected to light microscopy. Images were taken at 20X objective B. Immunofluorescence scans of 10 day fixed three-dimensional MDA-MB-231 breast cancer derived organoids, left untreated, or incubated with the TGFβ receptor inhibitor SB431542 (KI), TGFβ alone or together with the inhibitor, and subjected to PIAS1 (red) indirect immunocytochemistry and nuclei (blue) staining (Materials and Methods). Images were taken at 40X objective.
